# The epistemic roles of clinical expertise: An empirical study of how Swedish healthcare professionals understand proven experience

**DOI:** 10.1371/journal.pone.0252160

**Published:** 2021-06-02

**Authors:** Barry Dewitt, Johannes Persson, Lena Wahlberg, Annika Wallin

**Affiliations:** 1 Department of Engineering & Public Policy, Carnegie Mellon University, Pittsburgh, Pennsylvania, United States of America; 2 Division of Medical Ethics, Lund University, Lund, Sweden; 3 Department of Philosophy, Lund University, Lund, Sweden; 4 Department of Philosophy, Lund University Cognitive Science, Lund University, Lund, Sweden; 5 Department of Law, Faculty of Law, Lund University, Lund, Sweden; Nord University, NORWAY

## Abstract

Clinical expertise has since 1891 a Swedish counterpart in proven experience. This study aims to increase our understanding of clinicians’ views of their professional expertise, both as a source or body of knowledge and as a skill or quality. We examine how Swedish healthcare personnel view their expertise as captured by the (legally and culturally relevant) Swedish concept of “proven experience,” through a survey administered to a simple random sample of Swedish physicians and nurses (2018, *n = 560*). This study is the first empirical attempt to analyse the notion of proven experience as it is understood by Swedish physicians and nurses. Using statistical techniques for data dimensionality reduction (*confirmatory factor analysis* and *multidimensional scaling*), the study provides evidence that the proven experience concept is multidimensional and that a model consisting of three dimensions–for brevity referred to as “test/evidence”, “practice”, and “being an experienced/competent person”–describes the survey responses well. In addition, our results cannot corroborate the widely held assumption in evidence-based medicine that an important component of clinical expertise consists of experience of patients’ preferences.

## 1. Introduction

Is access to the best evidence from clinical trials and observational studies enough for healthcare professionals to make medical decisions? Most would answer in the negative, adding–in particular–that clinical expertise and judgment are also required. Evidence Based Medicine (EBM) acknowledges clinical judgment and expertise alongside the importance of clinical research (see e.g. [1–3]). In nursing, clinical judgment, in tandem with scientific evidence, is often said to be necessary to guide the healthcare professional in her decisions [4], and to be critical to “excellent patient care decisions and outcomes” ([5], p. 86). The Royal College of Nursing [6] even defines nursing as the use of clinical judgment in the provision of care.

There are several roles that clinical judgments and the expertise of health care personnel can play. These are often assumed to be epistemic and/or to harbor ethical content. The evidence-based medicine movement has stressed the *implementation* role of clinical expertise. The implementation role is important even when there is solid scientific evidence, because it is needed to think through how clinical trial results apply to practice and local settings. Here, practitioners consider how to tailor scientific evidence to the situation at hand (e.g. [2], p. 71 and [7–9]). Others have pointed to the *evidentiary* or *compensatory* role of clinical expertise, which occurs when the available scientific evidence is weaker or even irrelevant to the situation at hand (e.g. [10–12]). In the evidentiary role, clinical expertise is important in its capacity to harbor practice-derived evidence or personal experience which can be used in the absence of scientific evidence. This evidentiary or compensatory role is more difficult to acknowledge in EBM (see e.g. [9]) and has also been proposed as an overlooked characteristic of clinical judgment: “Lacking any formally identified methods of design and evaluation, most clinicians would use the term ‘clinical judgment’ when pressed to specify the methods, reasoning, and data used in clinical decisions” ([10], p. 799). Thirdly, evidence based medicine also emphasizes the important role of clinical judgment to identify and use *patients’ preferences* [2] as something that is central for decision-making but which scientific evidence cannot contribute. Whether a particular treatment is acceptable to the patient, and how it should be implemented therefore requires sound judgment. Neighbouring examples of how a patient perspective is integrated in clinical judgment and expertise in nursing are found in Tanner ([4], p. 204), who claims that “sound clinical judgment rests to some degree on… an engagement with the patient and his or her concerns”.

It is thus evident both from within and from the outside of the EBM-context that both scientific evidence *and* clinical expertise are needed for sound medical decision-making. We have a fairly good understanding of scientific evidence, at least as it is interpreted in medical discourse; i.e., what it is, how it is produced and accumulated over time, and how to judge its merits in a decision-making context. A prominent example is the Grading of Recommendations Assessment, Development and Evaluation (GRADE), which was developed with the ambition to provide “a common, sensible and transparent approach to grading quality (or certainty) of evidence and strength of recommendations” (https://www.gradeworkinggroup.org accessed 2020-06-16). Unfortunately, we have a much poorer understanding of the clinician’s expertise and its role in decision-making. We do not know what it is, and therefore we do not know how it accumulates over time, and how to judge its merits in decision-making contexts. Most importantly, we do not know how clinicians themselves view the role of their own expertise in relation to the medical literature, which together form the epistemological basis for the medical care they provide. The empirical study we describe below aims to answer that question, which is important because the clinicians’ own models of their knowledge and expertise will guide their medical practice, how medicine is taught, and how clinicians interpret the laws meant to regulate them.

Sweden has a long history of emphasizing the importance of clinical expertise and judgment in the legal regulation of health care. It is instantiated in the Swedish phrase *beprövad erfarenhet*, whose best English translation is, arguably, “proven experience.” Law requires that health care in Sweden meets the standards of *vetenskap och beprövad erfarenhet* ([13], 6:1 and [14], 1:7)–“science and proven experience”–and thus both concepts explicitly guide the training and legal regulation of healthcare providers. In the case of physicians, they have done so for over a century. Given the continued presence of a legal requirement of proven experience since 1891, Swedish medical professionals have a long history of thinking about the epistemic roles and the nature of clinical judgment in relation to the science of medicine, the skills and expertise of the individual practitioner, and the body of aggregated clinical or practical knowledge, making it a particularly interesting medical system to study. Sweden is not alone in this, of course. The notion of proven experience is used in other Nordic countries (e.g., Finland), and many jurisdictions define the threshold for good care by reference to clinical expertise and practice (e.g., [15]).

Its long history in the legal regulation of Swedish health care does not, however, mean that the notion of proven experience is well defined. This is the first empirical attempt to analyse the notion of proven experience as it is understood by Swedish physicians and nurses. By an empirical study of health care professions’ understanding of the notion, using statistical techniques for data dimensionality reduction, we not only develop a methodology for studying how clinicians understand concepts related to clinical expertise but also provide the first empirical analysis of how the notion of proven experience is understood by Swedish physicians and nurses.

Moreover, proven experience does not have to be studied as a proxy for clinical expertise; it has considerable value as a stand-alone construct. As we have seen, in comparison with EBM the Swedish construct allows for evidence that complements scientific evidence in a more straightforward way [9]. Making this construct more explicit would increase our understanding of changes in how clinicians conceptualize and integrate evidence over time and how this might differ between healthcare professions. Although it is not entirely clear how interprofessional communication is best improved, consensus appears to be that it is important for health outcomes [16]. Knowing more about how professions understand evidence could have practical implications for team communication and lifelong learning.

## 2. Previous work on clinicians’ views of proven experience

The present study was preceded by others, which informed its design:

a) A review of approximately 900 mentions of “proven experience” in the Swedish *Läkartidningen* (the Swedish Medical Association’s weekly publication) between 2006 and 2014 [17, 18].
b) A review of court judgments and opinions of medical experts in court decisions relating to Swedish patients’ right to reimbursement for expenses associated with health care in other European countries, where accordance with science and proven experience has been a condition for reimbursement [18, 19].
c) A review of Swedish governmental reports and governmental bills, and regulations and advice from Swedish health authorities using the term “proven experience” [20, 21].
d) Members of our team conducted interviews with medical professionals who have experience working with the Swedish Agency of Health Technology Assessments and Assessment of Social Services. The experts were informally asked about the relationship between science and proven experience, and the interviews provided us with sample statements on which we could base parts of the survey design. Interviews were conducted in Swedish, transcribed, and mapped on the dimensions suggested by the set of conceptual studies of proven experience performed by [17, 18]. Whenever a statement on proven experience did not easily fit into the conceptual framework, it prompted an addition to the questionnaire in order to probe for further, as yet not hypothesized dimensions. This should be seen as exploratory work.

Study (a) was qualitative, establishing six different ways “proven experience” is used and conceptual implications of each use. Studies (b) and (c) were also qualitative and confirmed the existence of three of the dimensions that were identified in Study (a). Study (b), (c) and (d) also contributed with additional real life expressions of the dimensions, and indicated the existence of other ways of use, not seen in study (a). We report the results from study (a) in detail here as they provide rationale for the way the present study was designed.

[17] reported that “proven experience” was often used in a way that implies it has an evidentiary nature, and is referred to in order to show or argue that something has been tested in clinical practice (see Table 1, dimension 1). For instance, some reported uses in that study built on a distinction between (mere) experience and proven experience in terms of how corroborated the experience was, indicating that proven experience, unlike personal experience, entails that the experience has been effectively tested:

“Patients should be able to rely on the fact that they will be diagnosed and treated in accordance with accepted practices that rest on solid ground. They have the right to expect to be treated in accordance with science and proven experience.”

([22], p. 2547, authors’ translation)

**Table 1:** Dimensions of proven experience according to [17, 1,8].

1: The seriousness of the test (i.e. the evaluation of the experience/practice)
2: The practice as origin of the experience
3: The practice as a mechanism for testing the experience
4: The practice as evidence
5: The amount/extent of an individual’s experience
6: The amount/extent of experience within a defined group

When the term “proven experience” is used in this manner, and emphasis is put on its capacity for testing and/or providing evidence for a treatment’s effectiveness, the epistemic role assigned to proven experience becomes similar, we argue, to that of science in providing reliable evidence for courses of action. For instance, proven experience has often been tested, but only in an informal way. As mentioned above, this evidentiary or compensatory role is difficult to acknowledge in EBM.

[17] also found that “proven experience” was sometimes used to refer to a quality that an individual or a team could possess–the quality of being experienced–rather than to the content of that experience:

“A person can be appointed as scientific advisor if (s)he has proven experience from relevant scientific fields”

(translated from the National Board of Health and Welfare’s webpage, accessed 2015-09-25, in [17])

“Many physicians, especially at the larger hospitals, are given scientific training, but do they get the proven experience they need? Do they have time for regular patient care, and follow-up, or are the patients too quickly sent back to an overburdened primary care?”

([23], p. 850, authors’ translation)

The use of proven experience as the quality of being experienced is kindred to the idea in EBM that clinical expertise and judgment is something that individual clinicians *acquire* through clinical experience and clinical practice [1].

A third way of using “proven experience” reported in [17] implied that a given treatment is widely spread in a particular community by being common or accepted practice (see Table 1, dimension 6). This use is seen in combination with the evidentiary role in the quote from Flodin above [22], but also occurs on its own:

“Proven experience refers to methods that are used in health care and believed to be effective. It includes what the medical profession regards as established practice”

([24], p. 198, authors’ translation)

Another example of proven experience as accepted practice is a paper in *Läkartidningen* discussing a decision from the Health and Social Care Inspectorate where a physician had been criticized for not acting in accordance with science and proven experience [25]. That paper contains objections to this decision as did ensuing comments from readers emphasizing that the physician had acted in accordance with common practice.

[17] also reported uses implying that proven experience has its origin in practical experience rather than in science or policy (see Table 1, dimension 2).

“Much of what is done within proven experience is good. One must not under-estimate the need for new technology. The opposite would be to introduce each new method at the same time and in the same way everywhere, which would lead to stagnation.”

([26], p. 15, authors’ translation)

The occurrences of “proven experience”–in the Swedish Medical Association’s weekly publication *Läkartidningen–*that [17] analysed thus shows that “proven experience” is used in conceptually rather different ways within (and when communicating with) the same community of users. In this and in a later study [18], it was suggested that at least six conceptual ‘dimensions’ of proven experience seemed to be present in the discourse, shown in Table 1.

Previous studies suggest that there are several perspectives one can take on proven experience. They do not, however, examine how these dimensions relate, how prominent they are, or whether and how they vary with different professions. This paper sets out to more closely examine how Swedish physicians and nurses understand “proven experience”. It does so through a survey sent to a true random sample of the populations of Swedish physicians and nurses. The survey instrument was designed with two guiding questions in mind: How do Swedish health care staff understand “proven experience”? How does this understanding depend on their profession (i.e., on being a physician or a nurse)?

For the present purposes we conjectured that the differences between dimensions 1, 3 and 4 in [17] were too subtle to be distinguished in a survey. Table 2 displays how the dimensions were simplified.

**Table 2:** Contracted dimensions, making up the *ABCD-model*.

A: test/evidence (dimensions 1, 3 and 4)
B: being experienced/competent (dimension 5)
C: accepted practice (dimension 6)
D: origin in practice (dimension 2)

Below, we first describe the survey sample and survey instrument in detail, followed by a description of the statistical methods used to analyze the data, including pre-specified models. We then present the results of the empirical analyses, including post-hoc analyses. We conclude by discussing what our results tell us about how clinicians view their own professional expertise.

## 3. Methods

### 3.1. Sample

The data analyzed below comes from a survey of Swedish healthcare professionals. Statistics Sweden (SCB)–the Swedish federal statistical agency–used the professional registries of Swedish physicians and nurses as the sampling frame. The registries from 2015 were used, which listed 33 618 physicians and 91 174 nurses. Seven hundred of each profession were randomly selected to receive an invitation to complete the survey.

### 3.2. Survey instrument

The survey consisted of mostly Likert-scale questions about participants’ beliefs and ideas around the concept of proven experience. They were adapted from actual use in the Swedish Medical Association’s weekly publication, *Läkartidningen* (for examples see Supporting information), court judgments and opinions of medical experts in court decisions, Swedish governmental reports and governmental bills, and regulations and advice from Swedish health authorities using the term “proven experience”, along with utterances from interviews of medical professionals who have experience working with the Swedish Agency of Health Technology Assessments and Assessment of Social Services. However, F3–F18 involve both interpretations and clarifications. As a consequence, there are many-to-many relations between question prompts and expressions in actual use. The survey was piloted by one of the authors who pre-tested the questionnaire on a small sample of Swedish healthcare professionals (nurses (5), occupational therapists (2), physicians (1)). They filled in a preliminary version of the questionnaire while thinking aloud, noting any confusion or discrepancies. The piloting led to slight changes of wording of the question prompts. Statistics Sweden also examined the questionnaire, leading to some additional slight changes of wording. Demographic information was also collected. The survey was administered in Swedish, and participants could choose to reply through mail or a web page. Respondents returned the questionnaires voluntarily. By replying consent was assumed, as approved by Lund University’s Regional Ethics Board. The survey in this paper was approved by Lund University’s Regional Ethics Board October 2017 (decision dnr 2017/428). The invitation contained a full description of participants’ rights and how data was to be handled. An English translation of the full survey (translation by the authors) is available in the Supporting Information. The questions used in this study are displayed in Table 3. These are the survey questions that asked explicitly about the characteristics proven experience might have.

**Table 3:** Question prompts. Note that the Swedish word for “question” is *fråga*.

Question label	Question, with a 5-point Likert scale response mode for agreement
F3	That there is proven experience of a treatment in the field of health care means that it has been carefully tested in the field of health care.
F4	That there is proven experience of a treatment in health care means that it has been shown to be effective in the field of healthcare.
F5	That there is proven experience of a treatment in health care means that a group of healthcare professionals together have reached the conclusion that it works.
F6	That there is proven experience of a treatment in health care means that it is widely accepted among healthcare professionals.
F7	That there is proven experience of a treatment in health care means that its origins lie in the daily activities in health care.
F8	That there is proven experience of a treatment in health care means that it does not violate medical ethics.
F9	That there is proven experience of a treatment in health care means that it has been used by many health care professionals.
F10	Healthcare professionals can have proven experience of carrying out a medical measure.
F11	That there is proven experience of a treatment in health care means that it works in the day-to-day activities in health care.
F12	That there is proven experience of a treatment in health care means that it is used by healthcare professionals for the current purpose.
F13	Proven experience is obvious to anyone who has a lot of experience in the profession.
F14	That there is proven experience in treatment means that it is based on the professional’s common sense.
F15	Proven experience in health care includes experience of what patients prefer.
F16	That there is proven experience of a treatment in health care means that it is used by successful medical units.
F17	That there is proven experience of a treatment in health care means that there is information documented about what has happened when it has been used.
F18	That there is proven experience of a treatment in health care means that it has been used in health care for a long time.

#### 3.2.1. Design of survey instrument

The survey instrument was designed to capture nuances in the actual use of “proven experience”. Most of these were chosen to reflect the dimensions discovered in [17] and used real life expressions of these dimensions seen in the subsequent studies referred to in section 2 above. In addition, some questions (F5, F8, F11, F15, F16 and F17) were added to capture additional aspects of proven experience that were reported in these later studies, or that came to our attention when designing the study. So, for example, the prompt “That there is proven experience of a treatment in health care means that a group of healthcare professionals together have reached the conclusion that it works” (F5) was added to account for the fact that the Swedish Board of Health and Welfare considers conclusions from consensus conferences to reflect proven experience, and the prompt “That there is proven experience of a treatment in health care means that it does not violate medical ethics” (F8) was added after comparison to one of the ways the phrase “proven experience” was used by the Swedish Board of Health and Welfare in its official statements and advice. The survey instrument could thus be used both to test the pre-specified conceptual model of proven experience, as well as to explore other possible meanings of the notion.

Before deciding on the question prompts’ final form we piloted the survey questions on a group of physicians and nurses, and slight changes were made. It should be noted that as the differences between some of the dimensions of the pre-specified model are rather fine-grained, when we find “proven experience” in an actual sentence, it sometimes captures more than one dimension (see, for example, the quote from Flodin above [22], “Patients should be able to rely on the fact that they will be diagnosed and treated in accordance with *accepted practices* that *rest on solid ground*”, our emphasis). This is true also for some of the question prompts of the survey instrument.

#### 3.2.2. Hypotheses

We connected the conceptual model described in the previous section, Tables 1 and 2, with the survey questions in Table 3 by connecting the questions to the dimensions from that model. That is seen in Table 4. Since some alternative uses of proven experience were added these were given additional tentative dimensions that we believed mirrored their content. We prioritized question prompts that were related to the hypothesized dimensions in Table 2, and thus the tentative dimensions are to be seen as exploratory work, with only one question prompt each.

**Table 4:** Question prompts mapped onto prespecified (A-D) and explorative (E-J) dimensions.

Dimension	Questions
A test/evidence	**F3, F4**, (F9, F16 and F17 are relevant as well but are preferably categorized respectively in dimensions C, J and H)
B being experienced/competent	**F10, F13, F14**
C accepted practice	**F6, F9, F12** (F5 and F16 are relevant as well but are preferably categorized respectively in dimensions I and J)
D origin in practice	**F7, F18**
E patient preferences	**F15**
F local knowledge	**F11**
G medical ethics	**F8**
H documentation	**F17** (see also A)
I negotiated expert group consensus	**F5** (see also C)
J used by successful units	**F16** (see also A and C)

Several hypotheses can be formulated:

The *maximal conceptual content hypothesis* states that each competent user understands and is prepared to use “proven experience” in accordance with each sentence F3-F18. The authors had no particular pre-specified model of this hypothesis in mind.

The *plural-dimension understanding hypothesis* states that each competent user understands and is prepared to use “proven experience” in accordance with more than one of the conceptual dimensions above. Based on our previous work [17–21, 27], we believe the most likely form of this multidimensional understanding of proven experience is in Table 2, *the ABCD- model* for short. It can be found in the first four rows of Table 2 and contains the dimensions test/evidence; being experienced; accepted practice and origin.

The ABCD model is sparse for being a plural-dimension understanding model (only 4 dimensions) and also far from including all the conceptual content expressed in F3-F18 (only 10 of 16 questions). However, it is completely grounded in previous results. The model contains the hypothesized dimensions, but not any of the exploratory questions found under E-F in Table 4. In order to investigate how these other question prompts relate to the more firmly grounded A-D dimensions, we constructed a few exploratory models (described below) that made intuitive sense to us. Given the exploratory nature of these models we have made no attempt at examining the fit of every possible model.

Finally, the *profession-dependence hypothesis* states that there is a difference between the professions (nurses and physicians) with regard to how well a model (such as the ABCD model) fits the answers of the respondents; specifically, that the fit will be better for the physicians than the nurses. We include this model, because the questions used were based primarily on previous studies of physicians, and we need to understand to what extent also different medical professions (e.g., nurses), that are guided by the same legal framework, differ in their understanding of proven experience.

## 4. Statistical analyses

The survey data was analyzed using two methods: *confirmatory factor analysis* and *multidimensional scaling*. Below, we briefly describe each method, including their specific implementation in this study.

### 4.1. Confirmatory factor analysis (CFA)

Confirmatory factor analysis (CFA) is a dimensionality-reduction technique that is useful for when researchers have a conjecture about how the survey questions are related to underlying concepts [28, 29]. CFA is a form of statistical modeling. It proceeds by taking as input the hypothesized structure of how the questions relate to underlying dimensions (called, generally, *latent factors*). CFA estimation then attempts to estimate a model with the specified structure from the observed data, and provides statistics to judge whether it is likely that the observed data was in fact generated from a process with the hypothesized structure. Like other models, it does not say whether a particular structure is correct, but does provide goodness-of-fit statistics.

Prior to our analysis of the data, we instantiated the conceptual hypotheses corresponding to the maximal and plural hypotheses as CFA models (see the first two rows and columns (1)-(3) in Table 5).

**Table 5:** The estimated models from CFA.

(1)	(2)	(3)	(4)	(5)	(6)	(7)	(8)	(9)	(10)
	Model name	Model specification	χ^2^	*df*	*p*	RMSEA	CFI	TLI	SRMR
**Pre-specified**	*M*^*16*^_*9*_	A = F3 + F4	193.22	148	0.007	0.033	0.987	0.979	0.053
		B = F10 + F13 + F14							
		C’ = F6 + F9 + F12 + F7 + F18							
		E = F15							
		F = F11							
		G = F8							
		H = F17							
		I = F5							
		J = F16							
	*M*^*10*^_*3*_^†^	A = F3 + F4	93.82	64	0.009	0.041	0.982	0.975	0.056
		B = F10 + F13 + F14							
		C’ = F6+ F9 +F12 + F7 + F18							
**Exploratory**	*M*^*11*^_*4*_	A = F3 + F4	122.23	78	0.001	0.045	0.974	0.963	0.059
		B = F10 + F13 + F14							
		C’ = F6+ F9 +F12 + F7 + F18							
		H = F17							
	*M*^*14*^_*7*_	A = F3 + F4	171.32	120	0.001	0.039	0.980	0.970	0.056
		B = F10 + F13 + F14							
		C’ = F6 + F9 + F12 + F7 + F18							
		E = F15							
		F = F11							
		H = F17							
		I = F5							
	*M*^*10*^_*3*_ (*fully constrained version)*	A = F3 + F4	144.56	94	0.001	0.044	0.969	0.971	0.070
		B = F10 + F13 + F14							
		C’ = F6+ F9 +F12 + F7 + F18							
**Algorithmically defined (via *k-*means on MDS configurations)**	*M*^*16*^_*3*_	a = F3 + F5 + F8 + F17	171.32	120	0.001	0.057	0.948	0.939	0.074
		b = F4 + F6 + F7 + F9 + F11 + F12 + F18							
		c = F10 + F13 + F14 + F15 + F16							
	*M*^*14*^_*3*_	a = F3 + F5 + F17	257.54	148	0.000	0.052	0.957	0.947	0.068
		b = F4 + F6 + F7 + F9 + F11 + F12 + F18							
		c = F10 + F13 + F14 + F15							

Column (1) indicates whether the models (the rows) were pre-specified, exploratory (post hoc), or defined via the algorithmic procedure described in the Methods. Column (2) shows the model labels that we use in the main text, and column (3) shows the model specification (i.e., how the survey questions relate to the underlying dimensions). Columns (4)-(6) show the model’s chi-square statistics with associated degrees of freedom and a *p*-value for the associated hypothesis test (where the null hypothesis is an exact model fit to the data). Columns (7)-(10) show the RMSEA, CFI, TLI, and SRMR fit indices, respectively.

^*†*^ Corresponds to the prespecified *plural-dimension understanding* model. Note two dimensions were collapsed into one because of estimation requirements (see the main text).

We estimated these models for all the data together, using a multiple group CFA procedure where we allowed all parameters to vary and where all the latent factors (the dimensions of interest) are correlated. That is equivalent to testing *configural invariance* [29, 30], that the physicians and the nurses each conceptualize the dimensions of proven experience similarly (i.e., according to the specified model structure). We used the **lavaan** package (v0.6–7) [31] in R (version 4.0.2) to estimate all CFA models. We tested the *profession-dependence hypothesis* by using an ANOVA to compare the configural invariance model with the model where all parameters across the two groups are forced to be equal, to see whether the fit is significantly different when there are more constraints on the parameters between the professions.

### 4.2. Multidimensional scaling (MDS)

Multidimensional scaling (MDS) is a data visualization technique for high-dimensional data [32]. In our case, it places survey questions on a plot whose dimension is chosen by the researcher, such that the (euclidean) distance between the questions is a proxy for how similar or dissimilar the questions are, based on the data. The index that defines “similarity” can vary; here, we examine linear correlations. The input to the MDS algorithm is thus the correlation matrix of the data.

MDS attempts to place the survey questions in a plot so that the pairwise distances between the questions capture the pairwise correlations from the correlation matrix. The difficulty of reproducing the similarity relationships expressed in the correlation matrix as euclidean distances in a plot depends on the structure of that correlation matrix. We use an *ordinal* algorithm that treats the correlation data as ordinal, so that the ranking of the correlations is preserved but not necessarily other characteristics of the data (cf. *interval*-scaled algorithms). We focus on 2-dimensional plots. Of course, any dimensionality is possible, but only 2- and 3-dimensional plots are easily interpretable. More dimensions necessarily leads to a better fit.

Below, we produce configuration plots of the questions using ordinal MDS. All analyses were run using the **smacof** package version 2.1 in R version 4.0.2 [33]. Initially, we ran the following MDS analysis:

- All questions from Table 3 with data only from physicians
- All questions from Table 3 with data only from nurses

We ran *Procrustean analysis* on the second and third configurations scalings. That analysis attempts to match two configurations via Procrustean transformations–those that do not alter the structure of the configuration–thereby determining their similarity. In addition, we used the results of the above pre-planned configurations and all of the CFA models to undertake exploratory MDS analyses, described below. If the Procrustean analysis showed that the nurses’ and physicians’ results were similar, then we planned to pool the data for exploratory analyses that were based on the MDS configurations; if it showed they were dissimilar, then we would keep them separate.

### 4.3. Post hoc and sensitivity analyses

Using the results of the above analyses, we then undertook exploratory analyses. The models estimated post hoc that are the most relevant to our discussion are described in detail in the Results, below (additional exploratory models appear in the Supplementary information). They are an extension of the ABCD model with an extra dimension (documentation) and a further extension with eight dimensions (ABCD + E, F, H, I) covering fourteen of the existing question prompts.

As a sensitivity analysis on our CFA results (both pre-specified and exploratory), we used the MDS results and *k-*means clustering [34] to choose alternative CFA models as purely algorithmic exploratory models. That is, *k-*means were used as a way to algorithmically specify CFA dimensions based on the correlations between questions rather than a theory of how the questions relate, allowing us to see how well our theory-driven (pre-specified and other exploratory) models compare to one chosen based purely on the data.

## 5. Results

305 nurses and 307 physicians completed the survey, a response rate of roughly 44% for each group. We removed participants who did not provide responses on all questions in Table 3 or who provided uninterpretable responses (e.g., selecting more than one response), which removed 25 physicians and 27 nurses, leaving a total sample of 560 participants (282 physicians and 278 nurses). Of those 282 physicians, 100 responded using the paper version of the survey and the remaining 182 used the online version of the survey. Of the 278 nurses, 120 responded by paper, and the remaining 158 using the online version. The Supplementary information contains demographic information on the samples: age, gender, and years of experience as a physician or a nurse.

**Fig 1:**
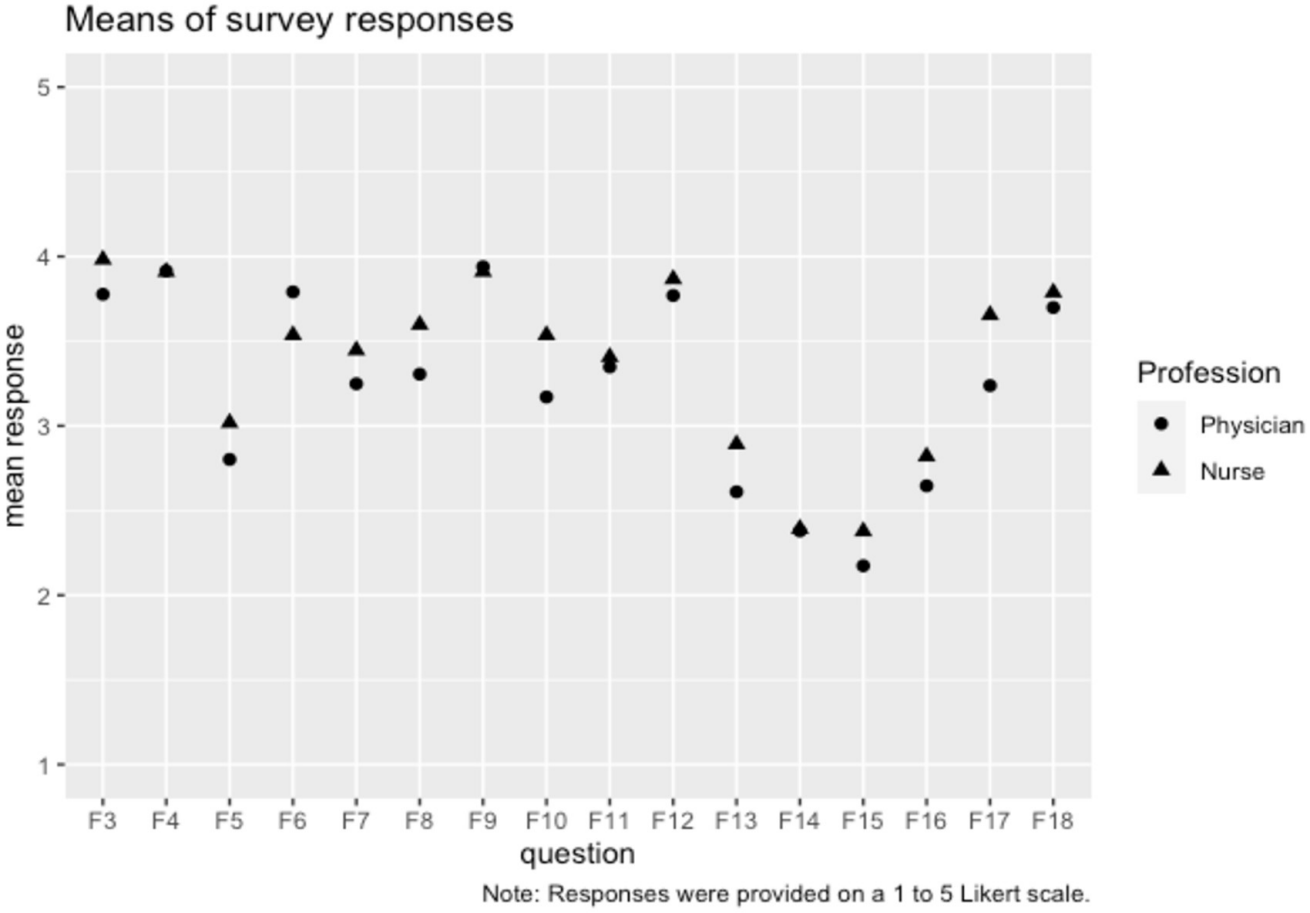
Means of survey responses for the physicians and the nurses.

Fig 1 shows the means of the responses to each question. The Supplementary Information contains the correlation matrix of the questions as well as contingency tables for each question (in separate excel document).

### 5.1. Confirmatory factor analysis

The CFA models are assessed through four fit statistics: root mean square error of approximation (RMSEA), comparative fit index (CFI), Tucker–Lewis index (TLI), and standardized root mean square residual (SRMR). Table 5 displays the fit statistics of the models described above. The model estimates are labeled *M*^*n*^_*m*_, where *n* is the number of questions used in the model and *m* is the number of dimensions. Conventional cuttoffs for “good” fit using the RMSEA, CFI, TLI, and SRMR indices are less-than 0.05, greater-than 0.95, greater-than 0.95, and less-than 0.08, respectively [29]. The model chi-square statistic, while reported, is less useful because of its sensitivity to sample size [28, 35]. Instead, we rely on the other four indices and do not privilege any single one.

Upon estimating the *ABCD-model* adapted from [17]–there was such a high correlation between dimensions (latent factors) C and D that we re-estimated the model combining those dimensions (otherwise, we were left with a model with a covariance matrix of latent factors that was not positive definite). Because dimensions C and D collapsed into one dimension, we will consequently refer to the conjunction of C and D as C’ (practice), and to the conceptual model suggested by the *estimate* of the ABCD model as the ABC’ model. That estimate of the pre-specified ABCD model, *M*^*10*^_*3*_, and in effect the *plural-dimension understanding hypothesis*, shows good fit to both physicians and nurses, with goodness-of-fit indices all below conventional cutoffs. Similarly, the *maximal conceptual content hypothesis* showed the same high correlation between factors; adjusting it to *M*^*16*^_*9*_ by collapsing the two dimensions (for the same reason we collapsed C and D above), we also have a good fit for both groups.

We then considered other ways in which question prompts not devised to test our original ABCD model could be combined meaningfully into dimensions. This led us to two other models.

One of the question prompts F17 (“That there is proven experience of a treatment in health care means that there is information documented about what has happened when it has been used”) was mentioned repeatedly by nurses, but not physicians in the interviews described in section 2. This is mirrored in Fig 1, where nurses give this specific question higher ratings than physicians. In addition, documentation and collegial sharing of experiences are key components of the stipulative definition of proven experience in the Swedish school system [36], making the dimension particularly interesting. We therefore gave our original ABC’ model an extra dimension, *documentation*. Adding the F17 question to the original model gave us *M*^*11*^_*4*_ and we note that it fits well when it is applied to both physicians and nurses (first of the exploratory models in Table 5).

To further examine the explorative dimensions (F-J), we constructed *M*^*14*^_*7*_ which includes questions concerning patient preferences, local knowledge, documentation and negotiated expert group consensus.

### 5.2. Multidimensional scaling

**Fig 2:**
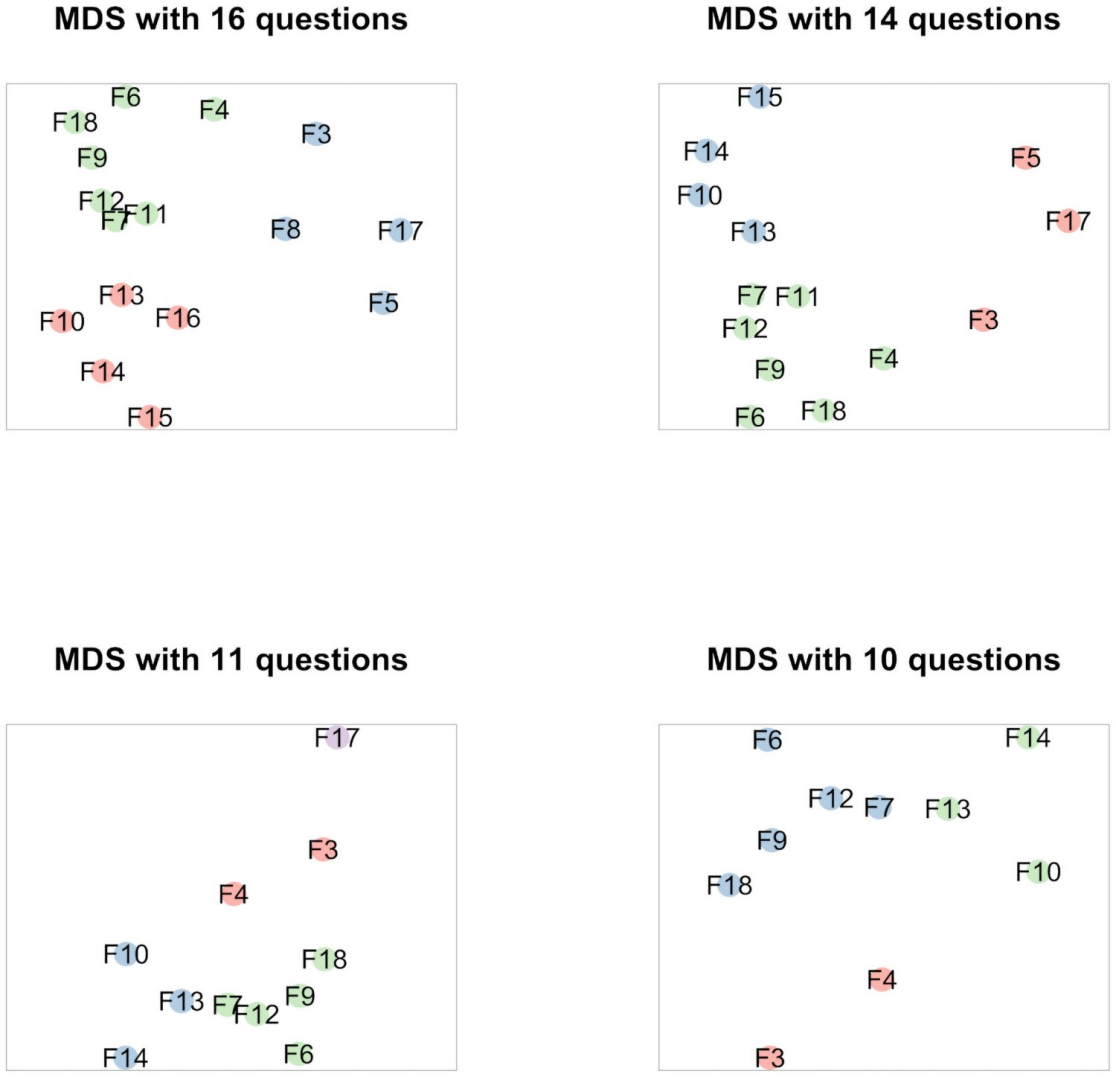
Configuration plots for the MDS solutions for all 16 questions, and then subsets of interest. Proximity of questions in the plots represents the correlation between responses, so that questions that are close received highly-similar responses. Colours correspond to groups of questions chosen through an application of k-means clustering.

We produced MDS configurations for the same sets of question prompts as the pre- and post-hoc models proposed and tested above. Thus, we produced configurations for all (16) question prompts and for the relevant subsets of 14, 11 and 10 questions. Here question prompts have no hypothesized relationship, but are rather plotted so that we examine how similar or dissimilar they are based purely on their empirical relationships, where that is measured using linear correlation as the index of similarity. The resulting configuration plots are seen in Fig 2, with colourings created via *k-*means. The plots were created using all of the data together, after a Procrustean analysis determined there were few differences between the plots of the nurses and physicians estimated separately (see Supplementary information). Information about the fit of the MDS solutions (Shepard plots etc.) is in the Supplementary Information.

From left-to-right in Fig 2, we see that the *k-*means algorithm chose three dimensions when considering the MDS configuration for all 16 questions, in contrast to our *maximal conceptual content hypothesis*. The k-means algorithm chose three dimensions also when considering the MDS configurations for 14 and 10 questions, but four dimensions for the configuration for 11 questions. The ways that the questions are grouped differ somewhat across the configurations. Interestingly, however, the question prompts that make up dimension B (*being experienced/competent*, prompts F10, F13 and F14) and dimension C’ (*accepted practice/origin in practice*, prompts F6, F9, F12, F7 and F18) in the ABC’ model, are grouped together across all configurations. Hence these two dimensions seem to be very robust. In both configurations (for 16 and 14 questions) where F15, the patient preference prompt, is included, it is grouped together with the prompts belonging to dimension B. The bringing together of the patient preference prompt with the prompts mirroring the quality of being an experienced person suggests an understanding quite similar to the notion of clinical expertise that can be found in the Evidence Based Medicine framework.

The question prompts in dimension A (*test/evidence*, prompts F3 and F4) are closely located in all configurations. However, the algorithm places them in different dimensions in the configurations for 14 and 16 questions, which indicates that dimension A is perhaps less robust than dimensions B and C’. In both configurations, question prompt F4 (…shown to be effective in the field of health care”) is grouped with the questions belonging to dimension C’ (practice), whereas question prompt F3 (“…carefully tested in the field of health care”) is grouped with question prompts F5 (“… a group of health care professionals together have reached the conclusion that it works”) and F17 (“there is information documented about what has happened when it has been used”). Potentially, F3, F5 and F17 all point to possible mechanisms that increase the likelihood that the proven experience is systematic and reliable. Such mechanisms are not present to the same extent in F4, which rather describes the outcome of a possible test (“shown to be effective”). This outcome-focus could also make F4 more similar to the C’-dimension (practice), in that it indicates that the treatment has been (successfully) implemented.

Finally, it is very interesting to note that the 11-question configuration (third panel of Fig 2) shows clusters of questions exactly describing the 11-question *post hoc* model *M*^*11*^_*4*_ described above, where documentation is given an extra dimension. Most importantly, the 10-question configuration proposes three dimensions that *are exactly the same* as the ABC’ model, estimated in the 10-question model *M*^*10*^_*3*_ instantiating and confirming the *plural dimension hypothesis*. The corresponding CFA models inspired by the clusters chosen through *k*-means are in the bottom rows in Table 5. Note that those for the 11- and 10-question models are omitted because they are exactly the same as the 11- and 10-question models that already appear in the table.

### 5.3. Model comparisons

We can also compare those models in Table 5 that have the same questions with an ANOVA. Table 6 shows those comparisons.

**Table 6:** ANOVAs comparing two pairs of nested models from Table 5.

(1)	***df***	**χ**^**2**^	**χ**^**2**^_**Δ**_ **(Δ*df*)**	***p***
*M*^*16*^_*9*_	148	193.22		
*M*^*16*^_*3*_	202	382.27	126.03 (54)	1.1e-07
(2)	***df***	**χ**^**2**^	**χ**^**2**^_**Δ**_ **(Δ*df*)**	***p***
*M*^*14*^_*7*_	120	171.32		
*M*^*14*^_*3*_	148	257.55	54.02 (28)	0.0022
(3)	***df***	**χ**^**2**^	**χ**^**2**^_**Δ**_ **(Δ*df*)**	***p***
*M*^*10*^_*3*_	64	93.823		
*M*^*10*^_*3*_ *(Fully constrained)*	94	144.56	32.77 (30)	0.33

The first ANOVA (panel (1)) in Table 6 compares the pre-specified model *M*^*16*^_*9*_ (third row in Table 5) instantiating the *maximal conceptual content model* to *M*^*16*^_*3*_, the second-to-last row of Table 5, which was defined algorithmically using the MDS procedure described above. It suggests that the model with more parameters (*M*^*16*^_*9*_) fits the data better than the model coming from the algorithmic MDS procedure. The second ANOVA (2) in Table 6 compares the *post hoc* exploratory model *M*^*14*^_*7*_ (sixth row of Table 5) and the algorithmically-defined *M*^*14*^_*3*_ (last row of Table 5). It also shows that the more complex model (*M*^*14*^_*7*_) has a better fit.

Given that the prespecified *ABC’ model* was constructed almost entirely from utterances in a discourse of physicians, we were surprised at the similar fits when the model was applied to nurses, especially since some of the added question prompts in the full model were taken directly from a nursing context. In fact, when we tested the configural invariance model against the model where all parameters are constrained to be equal (third-to-last row of Table 5), we found no difference (panel (3) in Table 6), indicating invariance of the model parameters across the physicians and nurses.

## 6. Discussion

Combining results from confirmatory factor analysis and multidimensional scaling, we find strong support for the *plural-dimension understanding*, instantiated in the ABC’ model adapted from Persson and Wahlberg (2015). First, the estimate of that model fits conventional cutoffs of the goodness-of-fit statistics for both physicians and for nurses (Table 5, row 3). Second, multidimensional scaling *independently* produces the *ABC’ model* (Fig 2). The model emphasizes three aspects of proven experience: testing and/or providing evidence, the quality of being experienced; and the status of being accepted/originating in practice. The last dimension (for brevity referred to as “practice”) is a merge of two dimensions (C and D, merged to C’) originally hypothesized as being separate.

We were surprised that dimensions C and D were so highly correlated while estimating the CFA model that we needed to merge them. They capture question prompts related to accepted practice and origin in practice respectively. It could be that both dimensions are seen as reflecting the fact that proven experience is associated with actual use. In retrospect, both appear to concern the issue of implementability: if a procedure comes from practice or is commonly accepted “on the floor”, issues of implementability are likely solved (in sufficiently similar contexts).

The fit of the ABC’ model is good both when allowing parameters to vary between the physicians and nurses and when they are forced to be equal (Table 6, panel (3)). The use of “proven experience” is similar within these professions, despite the fact that most of the utterances used in the survey came from a discourse central to physicians. To this we can add that dimensions A, B and our original dimension C have been found in Swedish court rulings and in official documents from the Swedish government and health authorities in official documents [19, 21]. The dimensions thus appear to be salient also in the legal framework surrounding medical practice in Sweden.

Our second prespecified maximal conceptual content model (*M*^*16*^_*9*_), which includes all question prompts, fares better than the reduced model (*M*^*16*^_*3*_) in the ANOVA comparison. Both models meet conventional cutoffs, but the number of dimensions in our proposed model makes it less useful than other proposed models, including the one derived from the MDS configuration (i.e., *M*^*16*^_*3*_). The model *M*^*16*^_*9*_ is hampered by our strategic consideration to add most question prompts for the dimensions that had already been proposed in the literature, allowing us to at least test these while not losing too many participants due to timing constraints. Moreover, the fact that the k-means algorithm chose only three dimensions when considering the MDS configuration for all 16 questions seems to contradict the maximal conceptual model–even though the ANOVA suggested that something is gained with the added complexity of *M*^*16*^_*9*_, it does not provide much in the way of improving our conceptual understanding.

Our results confirm the *plural dimension understanding hypothesis*. In particular, our pre-specified ABC’ model appears to be a useful way to map both physicians’ and nurses’ understandings of proven experience. Whereas, as mentioned above, the merged dimension C’ appears to concern implementability, dimension A in the model acknowledges proven experience as a mechanism for testing and providing evidence. Moreover, we speculated that the MDS results for 14 questions (*M*^*14*^_*7*_) indicate that participants cluster question prompts that address the need for proven experience to be reliable and systematic (seeing it as being carefully tested, negotiated expert consensus and being documented). We thus find evidence in our data for both of the implementation and evidence roles of proven experience. In future work, it would be useful to examine if how participants perceive proven experience also changes how they perceive its relationship to scientific evidence. Do, for instance, participants that give high ratings to proven experience with respect to careful testing, consensus and documentation also perceive it as being more similar to science, or perhaps as being equally important as science when making sound medical judgements. Does this vary between professions or even specialisations within a profession?

Interestingly, the agreement with question F15 (patient preferences) is the lowest among all the 16 question prompts relating to the dimensions (Fig 1), which suggests that experience of patient preferences is not seen as a substantial part of clinical expertise, despite the prominent role it plays in Evidence Based Medicine. This is, in one way, natural, given that patient preferences and clinical expertise are viewed as separate (but partly overlapping) components of Evidence Based Medicine, but on the other hand, clinical expertise is used to figure out how patient preferences can be met [2] and is therefore directed towards these preferences.

Staying with Fig 1, it can also be noted that the question prompts reflecting dimension B (being experienced/competent) tend to have slightly lower ratings than the question prompts reflecting dimensions A (test/evidence) and C’ (practice). This strengthens the argument that health care personnel read more into proven experience than would be expected if they simply adopted the notion of expertise from the Evidence Based Medicine movement, as “being experienced/competent” is the dimension most similar to the idea of clinical expertise within the movement. In particular proven experience appears to be most strongly linked to an epistemic role (where it can serve as evidence in its own right) as is witnessed by the dimension related to test / evidence, and to the dimension related to practice and in effect to issues of implementation, as witnessed by the merge of the C and D dimensions to C’.

As the *ABC’ model* was the most grounded in previous work, it is the only one for which we performed the comparison between the model allowing all parameters to vary between groups (the configural invariance model) and the fully constrained model (Table 6, (3)). As noted above, we observed no differences between physicians and nurses with respect to this model. An obvious next-step in the research process would be to do a full study of measurement invariance using the *ABC’* model and other models in order to reveal differences and similarities between how different healthcare professions (occupational therapists, dentists) conceptualize proven experience. That could lead to the development of a standardized questionnaire for assessing the healthcare professionals’ own models of their knowledge and beliefs, which would be valuable for improving care and education (e.g., understanding how other clinicians in other disciplines conceptualize evidence in order to improve team communication), for improving communication with medical experts in a legal setting, as well as for policy (e.g., ensuring epistemological statements in policy match the models of those regulated by them).

## 7. Conclusion

Clinical expertise is important in medical decision making and in the education of future medical professionals. But unlike scientific evidence, we have little understanding of what it means. Using the Swedish legal term “proven experience” as a proxy, we found that it has three fundamental dimensions: that it can function as test or evidence, that professionals acquire it (someone becomes experienced/competent) and that it originates from or is accepted in practice. This conceptualisation was shared by both physicians and nurses. The results in their own right, as well as the relationships between these dimensions, extend the amount and quality of empirical knowledge relating to this domain of medical epistemology. It allows for a type of knowledge that is not (usually) of the randomized controlled trial (RCT) variety but can do serious epistemic work: both by supporting or filling in for scientific evidence and by helping us apply relevant evidence to the concrete situations in health care.

## Supporting information

S1 File (DOCX)

S2 File (XLSX)
