# Supplementary material for: The epistemic roles of clinical expertise: An empirical study of how Swedish healthcare professionals understand proven experience
Source: PLoS One. 2021 Jun 2;16(6):e0252160. doi: 10.1371/journal.pone.0252160 (PMC8172027; doi:10.1371/journal.pone.0252160)
Supplement: S1 File — (DOCX) [file pone.0252160.s001.docx]

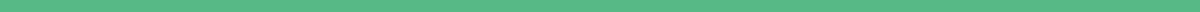


S1 File.

# OVERVIEW

Together with the excel document containing the contingency tables, this document provides supporting information for the manuscript, “The epistemic roles of clinical expertise: an empirical study of how Swedish healthcare professionals understand proven experience.”

First, we present the survey given to participants in the study. Second, we include a table showing the number of participants excluded from specific analyses. Third, we provide demographic information about the sample.

# Survey

Below is an English translation of the survey administered to the study’s participants. The English translation was created by the authors, but all participants took the survey in the original Swedish. The broader aim of the survey was to understand clinicians’ views of their own expertise, and in particular how they understood the legal notions “science and proven experience” governing their work. In this particular paper we target clinicians’ view of the legal role of the notion. The aggregate responses by physicians and nurses respectively to question prompts F3–F18 are provided in the corresponding excel document “Contingency Tables survey responses”.

The point of departure for the formulation of question prompts F3–F18 were expressions containing “proven experience” in actual use. However, F3–F18 involve both interpretations and clarifications of actual use. As a consequence, there are many-to-many relations between question prompts and expressions in actual use. *We will show it here by way of exemplification only*. Consider four of the quotations in the paper: The quote from Flodin (2011) can be interpreted in several related ways, disentangled in F3–F6; the quote from Linde (2010) can be interpreted as F7 and F10; Borgström (2007) uses the expression in a way that resonates with F6, F12, and F16; Borgström (2010) uses it in a way that is consistent with F3, F4, F7, F11, and F12. Instances of several of the question prompts can be found in the legislative history and other official documents relating to Swedish health law. For example, F8 can be found in Govt. Bill 1981/82:179 p. 9 and in IVO 2021. Variations of F6, F9, and F12 are expressed in Govt. Bill 1995/96:187 p. 33 and Govt. Bill 2002/03:50 p. 136. Expressions occurring in statements by the courts or by appointed experts in Swedish case law resonate with several of the prompts in the questionnaire, including F10 (Supreme Administrative Court 2004: 41), F17 (Gothenburg Administrative Court of Appeal 1616-06) and F18 (Stockholm Administrative Court 4046-17 and 4189-17). Ragnar Levi’s influential note on the webpage of Swedish Agency for Health Technology Assessment and Assessment of Social Services (SBU) [https://www.sbu.se/sv/publikationer/nya-vetenskap-och-praxis/vad-menas-med-beprovad-erfarenhet/] suggests, in addition to some of the earlier question prompts, the interpretations F13 and F14.

**Proven experience in communication between colleagues**

**A.** First two questions about how well the concept of "proven experience" works in the communication between colleagues.

**1. How certain do you feel that you know what the term "proven experience" means when you use the term?**

| 1 | 2 | 3 | 4 | 5 |
| --- | --- | --- | --- | --- |
| Not at all certain |  |  |  | Completely certain |

If you want to, please feel free to comment on your answer here: [Free text option]

**2. How certain do you think that your colleagues in healthcare generally feel about what the term "proven experience" means when they use the term?**

| 1 | 2 | 3 | 4 | 5 |
| --- | --- | --- | --- | --- |
| Not at all certain |  |  |  | Completely certain |

If you want to, please feel free to comment on your answer here: [Free text option]

**How do you perceive proven experience?**

**B.** In this section of the questionnaire there is a number of statements about proven experience. Please respond based on how you perceive proven experience, regardless of whether it is consistent with how you think others perceive the term.

**3. That there is proven experience of a treatment in the field of health care means that it has been carefully tested in the field of health care.**

| 1 | 2 | 3 | 4 | 5 |
| --- | --- | --- | --- | --- |
| Do not agree at all |  |  |  | Completely agree |

**4. That there is proven experience of a treatment in health care means that it has been shown to be effective in the field of healthcare.**

| 1 | 2 | 3 | 4 | 5 |
| --- | --- | --- | --- | --- |
| Do not agree at all |  |  |  | Completely agree |

**5. That there is proven experience of a treatment in health care means that a group of healthcare professionals together have reached the conclusion that it works.**

| 1 | 2 | 3 | 4 | 5 |
| --- | --- | --- | --- | --- |
| Do not agree at all |  |  |  | Completely agree |

**6. That there is proven experience of a treatment in health care means that it is widely accepted among healthcare professionals.**

| 1 | 2 | 3 | 4 | 5 |
| --- | --- | --- | --- | --- |
| Do not agree at all |  |  |  | Completely agree |

**7. That there is proven experience of a treatment in health care means that its origins lie in the daily activities in health care.**

| 1 | 2 | 3 | 4 | 5 |
| --- | --- | --- | --- | --- |
| Do not agree at all |  |  |  | Completely agree |

**8. That there is proven experience of a treatment in health care means that it does not violate medical ethics.**

| 1 | 2 | 3 | 4 | 5 |
| --- | --- | --- | --- | --- |
| Do not agree at all |  |  |  | Completely agree |

**9. That there is proven experience of a treatment in health care means that it has been used by many health care professionals.**

| 1 | 2 | 3 | 4 | 5 |
| --- | --- | --- | --- | --- |
| Do not agree at all |  |  |  | Completely agree |

**10. Healthcare professionals can have proven experience of carrying out a medical measure.**

| 1 | 2 | 3 | 4 | 5 |
| --- | --- | --- | --- | --- |
| Do not agree at all |  |  |  | Completely agree |

**11. That there is proven experience of a treatment in health care means that it works in the day-to-day activities in health care.**

| 1 | 2 | 3 | 4 | 5 |
| --- | --- | --- | --- | --- |
| Do not agree at all |  |  |  | Completely agree |

**12. That there is proven experience of a treatment in health care means that it is used by healthcare professionals for the current purpose.**

| 1 | 2 | 3 | 4 | 5 |
| --- | --- | --- | --- | --- |
| Do not agree at all |  |  |  | Completely agree |

**13. Proven experience is obvious to anyone who has a lot of experience in the profession.**

| 1 | 2 | 3 | 4 | 5 |
| --- | --- | --- | --- | --- |
| Do not agree at all |  |  |  | Completely agree |

**14. That there is proven experience in treatment means that it is based on the professional’s common sense.**

| 1 | 2 | 3 | 4 | 5 |
| --- | --- | --- | --- | --- |
| Do not agree at all |  |  |  | Completely agree |

**15. Proven experience in health care includes experience of what patients prefer.**

| 1 | 2 | 3 | 4 | 5 |
| --- | --- | --- | --- | --- |
| Do not agree at all |  |  |  | Completely agree |

**16. That there is proven experience of a treatment in health care means that it is used by successful medical units.**

| 1 | 2 | 3 | 4 | 5 |
| --- | --- | --- | --- | --- |
| Do not agree at all |  |  |  | Completely agree |

**17. That there is proven experience of a treatment in health care means that there is information documented about what has happened when it has been used.**

| 1 | 2 | 3 | 4 | 5 |
| --- | --- | --- | --- | --- |
| Do not agree at all |  |  |  | Completely agree |

**18. That there is proven experience of a treatment in health care means that it has been used in health care for a long time.**

| 1 | 2 | 3 | 4 | 5 |
| --- | --- | --- | --- | --- |
| Do not agree at all |  |  |  | Completely agree |

**Proven experience and other types of evidence**

**C.** In this part of the survey, there is a number of claims about how proven experience relates to other knowledge and evidence in health care.

**19.How important are each of the following types of knowledge for sound decision making in the healthcare sector?**

**Personal experience**

| 1 | 2 | 3 | 4 | 5 |
| --- | --- | --- | --- | --- |
| Not at all important |  |  |  | Very  important |

**Proven experience**

| 1 | 2 | 3 | 4 | 5 |
| --- | --- | --- | --- | --- |
| Not at all important |  |  |  | Very  important |

**Scientific evidence**

| 1 | 2 | 3 | 4 | 5 |
| --- | --- | --- | --- | --- |
| Not at all important |  |  |  | Very  important |

**20. How certain are each of the following types of knowledge in healthcare?**

**Personal experience**

| 1 | 2 | 3 | 4 | 5 |
| --- | --- | --- | --- | --- |
| Not at all certain |  |  |  | Completely certain |

**Proven experience**

| 1 | 2 | 3 | 4 | 5 |
| --- | --- | --- | --- | --- |
| Not at all certain |  |  |  | Completely certain |

**Scientific evidence**

| 1 | 2 | 3 | 4 | 5 |
| --- | --- | --- | --- | --- |
| Not at all certain |  |  |  | Completely certain |

**21. How systematic are each of the following types of knowledge in the healthcare system?**

**Personal experience**

| 1 | 2 | 3 | 4 | 5 |
| --- | --- | --- | --- | --- |
| Not at all systematic |  |  |  | Very systematic |

**Proven experience**

| 1 | 2 | 3 | 4 | 5 |
| --- | --- | --- | --- | --- |
| Not at all systematic |  |  |  | Very systematic |

**Scientific evidence**

| 1 | 2 | 3 | 4 | 5 |
| --- | --- | --- | --- | --- |
| Not at all systematic |  |  |  | Very systematic |

**The legal requirement of science and proven experience**

**D.** In this section of the survey, a few questions follow on how you perceive the requirement of science and proven experience in the legal regulation of healthcare.

According to the Patient Act, patients shall receive healthcare that is in accordance with science and proven experience. The Patient Safety Act states that healthcare professionals have a personal responsibility to carry out their work in accordance with science and proven experience.

**22. How certain do you feel that you know what the term "science and proven experience" means in the legal regulation of healthcare?**

| 1 | 2 | 3 | 4 | 5 |
| --- | --- | --- | --- | --- |
| Not at all certain |  |  |  | Completely certain |

**23. How do you feel about "science and proven experience" being used as quality requirement in the legal regulation of healthcare?**

| 1 | 2 | 3 | 4 | 5 |
| --- | --- | --- | --- | --- |
| Not at all satisfied |  |  |  | Very satisfied |

If you want to, please feel free to comment on your answer here: [Free text option]

**24. How do you feel about "proven experience" being used as quality requirement in the legal regulation of healthcare?**

| 1 | 2 | 3 | 4 | 5 |
| --- | --- | --- | --- | --- |
| Not at all satisfied |  |  |  | Very satisfied |

If you want to, please feel free to comment on your answer here: [Free text option]

**25. Who should - in your opinion - determine how the term "science and proven experience" should be interpreted in the legal regulation of healthcare?**

*You can choose more than one option if you want.*

Doctors and nurses and others in the healthcare professions

Courts / lawyers

SBU/Socialstyrelsen/IVO

Those who health care related research

Don’t know

If something else, please describe it here: [Free text option]

**Some questions about you**

**E.** At last,some questions about who you are.

**26. How old are you**

25 years or younger

26-30

31-35

36-40

41-45

46-50

51-55

56-60

61-65

66 years or older

**27. I am**

Woman

Man

I cannot / do not want to use the options above

**28. What are you currently employed as?**

Nurse

Doctor

Occupational therapist

If something else, please specify: [Free text option]

**29. If you have any specialization, which one is it?**

[Free text]

**30. How long have you had this employment?**

0-5 years

6-10 years

11-15 years

16-20 years

21-25 years

26-30 years

More than 30 years

**31. How long is it since you got your certification**

0-5 years

6-10 years

11-15 years

16-20 years

21-25 years

26-30 years

More than 30 years

**32. Where did you get your license?**

Göteborg

Köpenhamn

Lund

Stockholm

Umeå

Uppsala

Växjö

If something else, please specify: [Free text option]

**33. What academic degree do you have?**

[Free text option]

# Data and analysis

Participants were not required to answer every question. Thus, not every analysis presented in the manuscript used data from all 612 participants. Furthermore, because all participants were taking the same survey, differences between means of question responses were done using paired data. Thus, only those participants who answered both questions in a pair could be included. See Table 1.

| **Calculation** | **Number of participants excluded (*n* = 612)** |
| --- | --- |
| Mean of Q1 | 3 |
| Mean of Q2 | 5 |
| Mean of Q22 | 6 |
| Mean of Q23 | 8 |
| Mean of Q24 | 13 |
| Mean difference between Q1 and Q2 | 5 |
| Mean difference between Q1 and Q22 | 9 |
| Mean difference between Q2 and Q22 | 11 |
| Mean difference between Q23 and Q24 | 16 |

Table 1: Participants excluded from calculations used in the manuscript, because they did not have complete data for that calculation.

# Demographic information of the sample


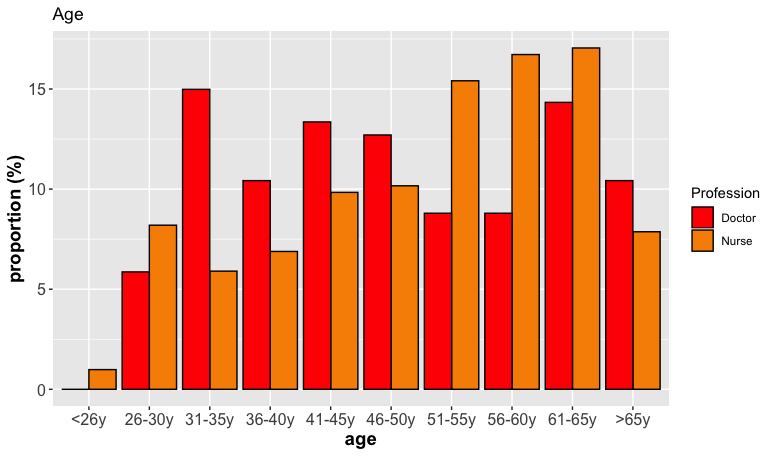


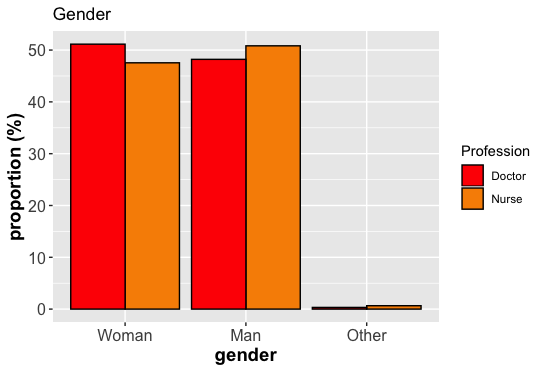


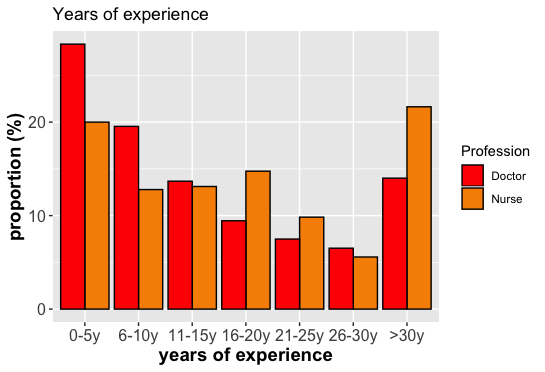


References:

Governmental Bill 1981/82:172 with prohibition on female circumcision.

Health and Social Care Inspectorate 2021, Dnr. 3.6.1-28734/2020-15, 2021-03-17.

Governmental Bill 1995/96:187. Patient injury act.

Governmental Bill 2002/03:50. Ethical review of research.

Supreme Administrative Court 2004: 41, RÅ 2004 ref. 41.

Gothenburg Administrative Court of Appeal 1616-06, 2008-03-07.

Stockholm Administrative Court 4046-17, 2017-05-15.

Stockholm Administrative Court 4189-17, 2017-05-15.
